# High Expression of *TTYH3* Is Related to Poor Clinical Outcomes in Human Gastric Cancer

**DOI:** 10.3390/jcm8111762

**Published:** 2019-10-23

**Authors:** Subbroto Kumar Saha, Polash Kumar Biswas, Minchan Gil, Ssang-Goo Cho

**Affiliations:** Department of Stem Cell and Regenerative Biotechnology, Incurable Disease Animal Model & Stem Cell Institute (IDASI), Konkuk University, 120 Neungdong-ro, Gwangjin-gu, Seoul 05029, Korea; subbroto@konkuk.ac.kr (S.K.S.); polashbiswas71@gmail.com (P.K.B.)

**Keywords:** TTYH3, gastric cancer, patient survival, clinical outcomes, cancer progression, multiomics analysis

## Abstract

Ion channels play important roles in regulating various cellular processes and malignant transformation. Expressions of some chloride channels have been suggested to be associated with patient survival in gastric cancer (GC). However, little is known about the expression and function of *TTYH3*, a gene encoding a chloride ion channel, in cancer progression. Here, we comprehensively analyzed the expression of *TTYH3* and its clinical outcome in GC using publicly available cancer gene expression and patient survival data through various databases. We examined the differences of *TTYH3* expression between cancers and their normal tissues using the Oncomine, UALCAN, and GEO (Gene Expression Omnibus) databases. *TTYH3* expression was investigated from immunohistochemistry images using the Human Protein Atlas database. Copy number alterations and mutations of *TTYH3* were analyzed using cBioPortal. The co-expression profile of *TTYH3* in GC was revealed using Oncomine. The gene ontology and pathway analyses were done using those co-expressed genes via the Enrichr tool to explore the predicted signaling pathways in GC. *TTYH3* mRNA and protein levels in GC were significantly greater than those in normal tissue. Kaplan–Meier analysis revealed the upregulation of *TTYH3* expression, which was significantly correlated with worse patient survival. Collectively, our data suggest that *TTYH3* might be a potential prognostic marker for GC patients.

**^+^** These authors contributed equally to this study.

## 1. Introduction

Deaths due to cancers are an increasing threat to human survival [1,2]. A total of 17.2 million incidences of cancer cases and 8.9 million cancer deaths were reported worldwide in 2016 in the Global Burden of Disease study [2]. Gastric cancer (GC)/stomach cancer (SC) is one of the most common causes of cancer death, with 1.2 million cases and 834.000 deaths globally in 2016 [2]. There have been improvements in the early detection and treatment of GC/SC, which has improved patient survival. Yet, GC/SC still causes many deaths. To improve patient survival in GC/SC, identification of novel therapeutic targets is crucial. Expression of genes are altered due to the accumulated genetic alterations or epigenetic modifications in all types of cancers. The profiles of differentially expressed genes (DEGs) in each cancer tissue reflects the cancer characteristics that are closely related to patient prognosis. DEGs associated with patient survival in GC/SC may be possible markers for early diagnosis and may be therapeutic targets. Determining this requires an understanding of the related mechanisms of cancer progression and aggressiveness.

Ion channels are transmembrane proteins that regulate the flux of ions through plasma membranes that are positioned between extracellular and intracellular spaces. Based on the types of ions that the channels permit the movement of, the channels are classified as calcium (Ca^2+^) channels, potassium channels, sodium channels, proton channels, nonselective cation channels, and chloride channels [3,4]. Ion channels have important roles in regulating various cellular processes underlying animal development and tissue homeostasis. Altered levels of ion channel expression as well as their activity are often associated with aggressive phenotypes of cancers, such as proliferation, apoptosis, migration, and drug resistance [3,4,5,6]. Various types of ion channels have been suggested as prognostic markers and/or therapeutic targets in GC/SC [7]. For example, potassium channel, KCND2, and calcium channel TRPV2 are correlated with clinical outcome of gastric cancer patients [8,9]. Cytosolic chloride ion has roles in GC/SC progression by regulating lysosomal acidification and autophagy functions [10]. Expressions of a few chloride channels, including chloride intracellular channel 1 (CLIC1) and Ca^2+^-activated Cl−channel transmembrane protein 16A (TMEM16A), have been suggested to be negatively associated with patient survival in GC [11,12]. The relationship between ion channels and prognosis in GC might motivate the study of other existing ion channel responsive genes which have yet to be studied.

The tweety family of genes (TTYHs) is reported as chloride channel responsive genes and has been contributing in several cellular processes including cell adhesion, cell division, tumorigenesis, and regulation of calcium activity [13]. The *TTYH1* gene, the first member of the TTYHs, encodes a protein that forms swelling-activated chloride ion channels [14]. *TTYH2* is the second member of the TTYHs family which was overexpressed in cancers [15,16]. *TTYH3* (tweety family member 3) is the third member that encodes one of three mammalian tweety-homologs (TTYH) that harboring chloride channel activities [16,17]. *TTYH3* is also known as a large-conductance Ca^2+^-activated chloride channel. Human *TTYH3* mRNA is mainly expressed in excitable tissues, including the brain, heart, and skeletal muscles [17]. However, little is known about the expression and function of the TTYHs in any cancer, although the expression of *TTYH2* was reported to be involved in cell proliferation and aggregation in colon carcinoma [15] and in the invasion and migration of osteosarcoma cells [18]. However, to the best of our knowledge, TTYH genes have not yet been elucidated using data mining tools. Therefore, this is the first data mining study to predict the possible role of *TTYH3* in gastric cancers as it is the only member of the TTYHs which was highly expressed in gastric cancer, based on publicly available gene expression and clinical data.

Given the fact that elevated expression of *TTHY3* in GC/SC multiple expression datasets, we undertook a comprehensive analysis to investigate the expression pattern of the *TTYH3* gene and its clinical outcome in GC/SC patients using numerous publicly available expression and patient survival datasets from various online platforms. In addition, we also analyzed genes that were co-altered with *TTYH3* in GC/SC that might be relevant in *TTYH3*-associated mechanisms in GC/SC progression and prognosis. The collective data provide supportive evidence for the use of *TTYH3* as a potential prognostic biomarker of GC/SC therapeutics.

## 2. Materials and Methods

### 2.1. Analysis of TTYH3 Expression in Various Cancers

The mRNA expression levels of *TTYH3* in various cancers and their normal tissue counterparts were analyzed using the Oncomine database (https://www.oncomine.org/resource/login.html) [19,20], Gene expression Profiling Interactive Analysis 2 (GEPIA2) (http://gepia2.cancer-pku.cn/#index) [21], and the Gene Expression across Normal and Tumor tissue (GENT) database (http://medical-genome.kribb.re.kr/GENT/) [22]. In GEPIA2, *TTYH3* expression of tumor samples in The Cancer Genome Atlas (TCGA) were compared to combined expression data of normal adjacent mucosa in TCGA and normal healthy stomach in Genotype-Tissue Expression (GTEx) [23]. *TTYH3* queries were carried out with default settings to obtain their respective expression pattern in all analyses with these databases.

### 2.2. Analysis of TTYH3 Expression in GC/SC and Its Normal Tissue

*TTYH3* mRNA expression in GC and normal counterparts was examined in the Oncomine database, the Gene Expression Omnibus (GEO) database (https://www.ncbi.nlm.nih.gov/geo/) [17], and the UALCAN web (http://ualcan.path.uab.edu/index.html) [24]. In the Oncomine database, the fold-change in mRNA expression in GC tissue compared to the normal tissue was obtained using the parameters of *p*-value < 1E−4, fold-change > 2, and gene ranking in the top 10%. Microarray datasets (Gene Series Expression, GSE) of accession numbers GSE27342 and GSE13911 were downloaded from the GEO database. Normalized raw transcriptome data were subsequently analyzed to evaluate the relative expression of *TTYH3* in GC relative to normal gastric tissue. Expression of the TTYH3 protein in GC and normal gastric tissue was investigated in immunohistochemistry images retrieved from the Human Protein Atlas database [25].

### 2.3. TTYH3 Gene Expression and Promoter Methylation Analysis in Each Clinical Characteristic with Data from TCGA

*TTYH3* mRNA expression and promoter methylation in each GC patient characteristic were examined in TCGA datasets using the UALCAN web with default settings [24]. *TTYH3* mRNA expression in cancer was separately analyzed with patient characteristics of sample types, individual cancer stage, age, histological subtype, race, gender, *Helicobacter pylori* infection status, and tumor grade compared to the normal gastric tissue expression. We also analyzed *TTYH3* expression in each clinicopathological parameters of GC patient using TCGA data via UCSC (University of California, Santa Cruz) Xena presented in Table 1 [26,27]. The statistical analysis between two variables was performed by unpaired t-test, and one-way ANOVA analysis was performed for more than two variables. Promoter methylation was analyzed according to individual cancer stage, age, race, gender, and tumor grade. Data from probes cg06316830, cg00798876, cg11076555, cg15755662, cg20199792, cg26540931, and cg19506025 in Infinium Human Methylation 450K chip was used for the promoter methylation data in UALCAN web.

### 2.4. Evaluation of Mutations and Copy Number Alterations (CNAs) of the TTYH3 Gene in GC/SC

We analyzed the mutations and CNAs of the *TTYH3* gene using the cBioPortal web (http://www.cbioportal.org/) [28,29]. The location and frequency of the mutations were estimated from samples from six studies available for SC in cBioPortal. Somatic CNAs were generated from RNA-seq data by the GISTIC (genomic identification of significant targets in cancer) algorithm with default settings and plotted with mRNA expression data using cBioPortal web. Correlation statistics was performed using Graph Pad Prism 7.0. The statistical analysis between two variables was performed by unpaired t-test, and one-way ANOVA analysis was performed for more than two variables.

### 2.5. Evaluation of the Relationship Between TTYH3 Expression and Patient Survival in GC/SC

The clinical relevance of *TTYH3* expression in GC/SC was investigated with the Kaplan–Meier plotter (http://kmplot.com/analysis/) [30] web-based tool by concatenating TCGA, GEO, and European Genome-phenome Archive clinical data with mRNA expression levels. This platform was used to quickly confirm disease prognosis, including overall survival (OS), post-progression survival (PPS), and first progression (FP). The *TTYH3* expression of each risk group was graphed into survival plots, and the prognostic index of each sample was estimated by Cox survival analysis. We generated survival curves with data of all patients or clinicopathological subgroups by dividing cohorts with auto-select best-cutoff mode. All other settings were default. Integrated meta-analysis to achieve an overall assessment of the clinicopathological significance of *TTHY3* in GC/SC from element GSE datasets included in Kaplan–Meier plotter web and patient survival results to conduct an integrated meta-analysis. We also performed univariate and multivariate survival analyses through Kaplan–Meier plotter web presented in Table 2. Statistical significance was evaluated by Kruskal–Wallis, Mann–Whitney, Fisher exact test, Mantel–Cox log rank, or Cox regression tests (*p < 0.05; **p < 0.01; ***p < 0.001).

### 2.6. Profiling of Genes Co-Expressed with TTYH3 

The co-expression profile of the *TTYH3* gene was analyzed using the Oncomine database. The co-expression profile identified *nexin-8* (*SNX8)* as the top positively correlated gene in GC. The gene co-expression profiling in GC/SC tissue was obtained from Oncomine web. We confirmed this positive correlation between *TTYH3* and *SNX8* transcript levels by analyzing GC patient data by drawing a heatmap using the TCGA database through the UCSC Xena web (http://xena.ucsc.edu/) [26,27].

### 2.7. Signaling Pathway and Gene Ontology (GO) Analyses of TTYH3 and Co-Expressed Genes

To find the pathways and GO shared by *TTYH3*-correlated genes from the Oncomine database, we used the Enricher web tool (https://amp.pharm.mssm.edu/Enrichr) [31]. The enriched GO and pathways were visualized as a bar diagram (Figure 8).

## 3. Results

### 3.1. TTYH3 mRNA Expression in Various Cancers

**Figure 1:**
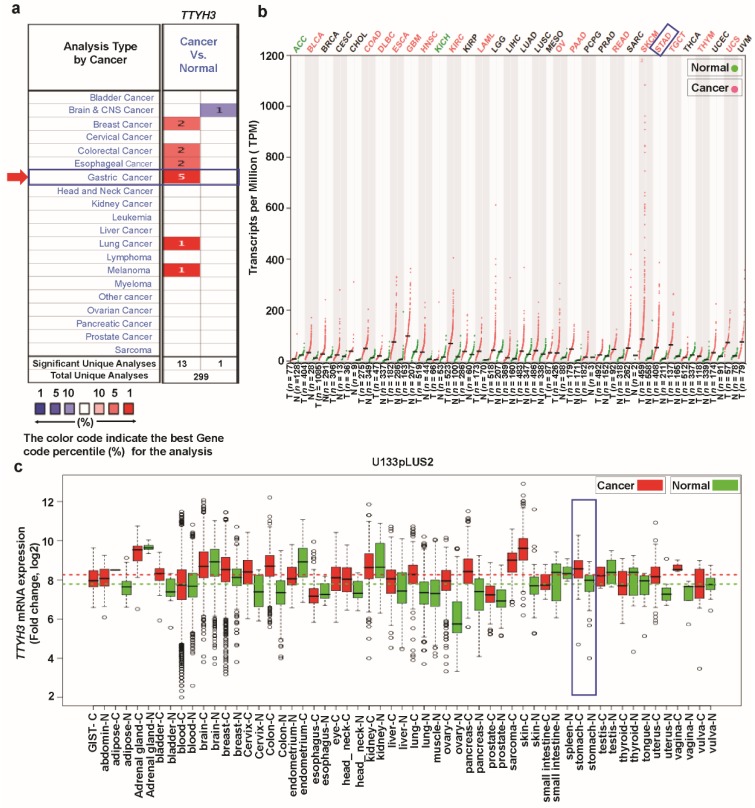
*TTYH3* mRNA expression in various cancer types: (**a**) The comparison indicated the number of datasets with *TTYH3* mRNA overexpression (left column, red) and underexpression (right column, blue) in cancers versus normal tissues. This graphic presentation originated from the Oncomine database (available at https://www.oncomine.org/resource/login.html), and the threshold was designed with the following parameters: *p*-value of 1E−4, fold-change of 2, and gene ranking of 10%. (**b**) The expressions of *TTYH3* in 33 types of human cancer in data from The Cancer Genome Atlas through GEPIA2 (Gene expression Profiling Interactive Analysis 2) web (available at https://gepia2.cancer-pku.cn): The gene expression profile across all tumor samples and paired normal tissues is shown as a dot plot. Each dot represents expression of samples. (**c**) Expression pattern of *TTYH3* mRNA in tumor and corresponding normal tissue: Data concerning *TTYH3* mRNA expression in various types of cancer were retrieved from the GENT (Gene Expression across Normal and Tumor tissue) database (available at http://medical-genomics.kribb.re.kr/GENT/). Boxes represent the median and the 25th and 75th percentiles. Dots represent outliers. Red boxes represent tumor tissues, and green boxes represent normal tissues. Red and green dashed lines represent the average expression value of all tumor and normal tissues, respectively.

To explore the expression pattern of *TTYH3* in various types of cancers, we examined the differences of *TTYH3* expression between the cancers and their normal tissues using three independent bioinformatics databases. In the Oncomine database, the number of significant unique analyses showing differences of mRNA expression in cancer tissue compared to the normal tissue was obtained with the parameters of *p*-value < 1E−4, fold-change > 2, and gene ranking in the top 10%. The comparison of expression level between each type of cancer vs. normal counterpart revealed the upregulation of *TTYH3* in gastric, breast, colorectal, esophageal, and lung cancers and in melanoma (Figure 1a). The increase of *TTYH3* in GC/SC was greatest. Only one analysis revealed the downregulation of *TTYH3* expression in brain and central nervous system cancer (Figure 1a). We further analyzed the expression of *TTYH3* between 33 types of human cancer and their normal tissues with the expression data retrieved from combined TCGA and GTEx data using GEPIA tools (Figure 1b). Among the 33 cancer types, 16 displayed significantly higher *TTYH3* expression levels compared to their normal counterparts and two cancers displayed lower *TTHY3* expression levels. In the GENT database, analyzed using the U133Plus2 platform, *TTYH3* expression was upregulated in certain cancer types, including bladder, breast, colon, lung, pancreatic, stomach, ovarian, and testicular cancers (Figure 1c). The data revealed the significantly increased expression of *TTYH3* in various cancer types. Expression levels of *TTYH3* in GC/SC were significantly higher in all three databases compared to their normal tissue (Figure 1c, denoted by the blue box).

### 3.2. TTYH3 mRNA and Protein Expression in GC/SC

**Figure 2:**
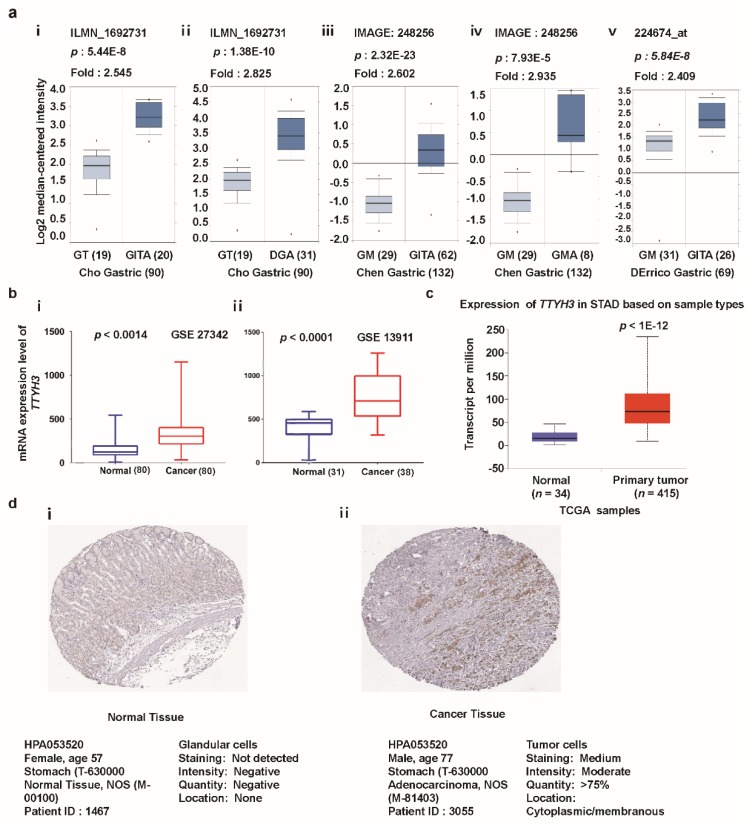
*TTYH3* expression is significantly upregulated in gastric cancer (GC)/stomach cancer (SC) tissue. (**a**) Box plot comparing specific *TTYH3* expression in normal (left plot) and cancer tissue (right plot) was derived from the Oncomine database. The fold-change of *TTYH3* expression in various types of GC/SC was determined using the Oncomine database. The data are gastric adenocarcinoma relative to normal gastric tissue (GT): Gastric Intestinal-Type Adenocarcinoma (GITA) relative to GT (i), Diffuse Gastric Adenocarcinoma (DGA) relative to GT (ii), GITA relative to normal Gastric Mucosa (GM) (iii), Gastric Mixed Adenocarcinoma (GMA) relative to GM (iv), and GITA relative to normal GM (v). The threshold was designed using the following specific parameters: *p*-value = 1E−4, fold-change = 2, and gene rank 10% (**b**) The mRNA expression of *TTYH3* was examined from the Gene Expression Omnibus (GEO) public database under accession numbers GSE27342 and GSE13911. (**c**) Expression of the *TTYH3* gene in The Cancer Genome Atlas (TCGA) database: Box plots showing the *TTYH3* mRNA expression in GC tumors (red plot) and their normal (blue plot) tissues was derived through ULCAN (http://ualcan.path.uab.edu/index.html). (**d**) The representative protein expression of TTYH3 in GC tissue (adenocarcinoma) and normal tissue (glandular cells) from the immunohistochemistry data from the Human Protein Atlas Project (http://www.proteinatlas.org/). Abbreviations: GT = Gastric Tissue, DGA = Diffuse Gastric Adenocarcinoma, GM = Gastric Mucosa, GITA = Gastric Intestinal Type Adenocarcinoma, GMA = Gastric Mixed Adenocarcinoma.

To observe the expression of *TTYH3* in various subtypes of GC/SC, we analyzed each individual subtype dataset from the Oncomine database (Figure 2a). Augmented expression of *TTHY3* was observed in datasets with diffuse adenocarcinoma, gastric adenocarcinoma, gastric mixed adenocarcinoma, and gastric intestinal-type adenocarcinoma. The data acquired from GEO datasets with accession numbers GSE27342 [32,33] and GSE13911 [34] also revealed the significant augmentation of mRNA expression in GC/SC compared to their normal counterparts (Figure 2b). Increased expression of *TTYH3* in GC/SC was also confirmed in the TCGA dataset using the UALCAN tool (Figure 2c). We next sought to verify this trend at the protein level between glandular cells (normal healthy tissue) and stomach adenocarcinoma (tumor tissue). In the immunohistochemistry data from the Human Protein Atlas project, 2 of the total 11 GC/SC patients’ samples had moderate or weak staining signals, whereas normal glandular cells in healthy stomach did not showed detectable *TTYH3* expression (see Figure 2d (i and ii)). Overall, expression data in multiple databases suggested that *TTYH3* expression could be augmented in GC/SC tissues compared to normal counterparts.

### 3.3. Association between TTYH3 Expression and Clinical Characteristics of GC/SC Patients 

**Figure 3:**
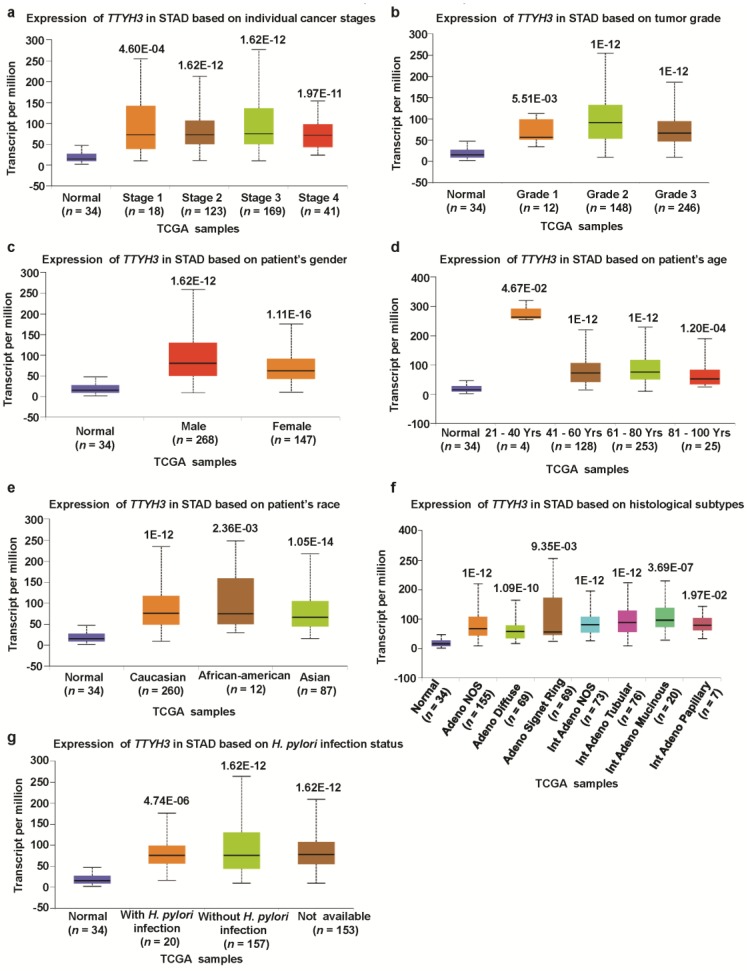
Association between *TTYH3* expression and clinical characteristics of GC/SC patients: The *TTYH3* mRNA expression level was expressed by box plots using UALCAN web (http://ualcan.path.uab.edu/index.html) for the patient characteristics of (**a**) individual cancer stages, (**b**) tumor grade, (**c**) patient gender, (**d**) patient age, (**e**) patient race, (**f**) histological subtypes, and (**g**) *Helicobacter pylori* infection. Abbreviation: STAD, Stomach Adenocarcinoma.

**Figure 4:**
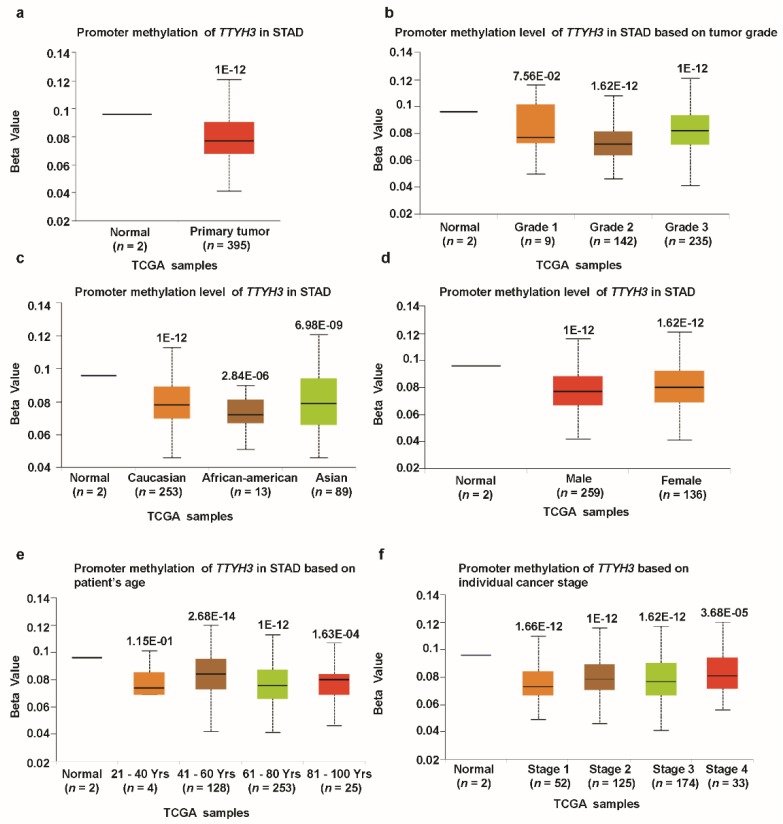
Promoter methylation of the *TTYH3* gene is significantly downregulated in GC/SC tissue (TCGA data). Promoter methylation levels of the *TTYH3* gene expressed as box plots from TCGA clinical data according to categorized GC patient characteristics using the UALCAN web tool: (**a**) Promoter methylation of *TTYH3* in GC tumor (different color plot) and their normal (blue plot) tissues based on (**a**) normal vs. primary tumor, (**b**) tumor grade, (**c**) patient race, (**d**) patient gender, (**e**) patient age, and (**f**) individual cancer stage. The beta value indicates the level of DNA methylation ranging from 0 (unmethylated) to 1 (fully methylated).

We investigated the association between *TTYH3* mRNA expression and the clinicopathological characteristics of GC/SC using TCGA data through UALCAN and UCSC Xena tools. Compared to the normal tissue, expression of *TTHY3* was augmented regardless of cancer stage (S1, S2, S3, and S4), tumor grade (G1, G2, and G3), gender (male and female), age (20–40, 41–60, 61–80, and 81–100 Yrs), race (Caucasian, African-American, and Asian), histological subtype, and *Helicobacter pylori* infection (Figure 3, Table 1). Interestingly, *TTYH3* expression was most enhanced in the early age group (21–40 years old) of GC/SC patients as compared to any other age group (Figure 3d). In terms of tumor grading, TTYH3 expression was significantly upregulated in all tumor grade (Figure 3b). *TTYH3* mRNA expression was also upregulated in all histological subtypes of GC/SC patients compared to their normal counterparts (Figure 3f). Other clinicopathological parameters including surgical approach, pharmaceutical therapy, radiation therapy, and targeted molecular therapy were also significantly correlated with *TTYH3* expression in GC/SC patients (Table 1). Promoter methylation is one of the essential epigenetic regulatory factors of gene expression. Promoter methylation was significantly reduced in GC/SC tumors compared to normal tissue counterparts in the UALCAN analysis of TCGA data (Figure 4a). The level of promoter methylation was also reduced regardless of patient characteristics, including cancer stage, tumor grade, gender, age, and race (Figure 4b–4f). Comparison between *TTYH3* expression and DNA methylation status suggested that the gene expression might be negatively related with some CpG sites. Overall, these data suggested the increased mRNA expression of *TTYH3* and reduced promoter methylation in GC/SC.

### 3.4. Mutations and CNAs of TTYH3 Gene in GC

**Figure 5:**
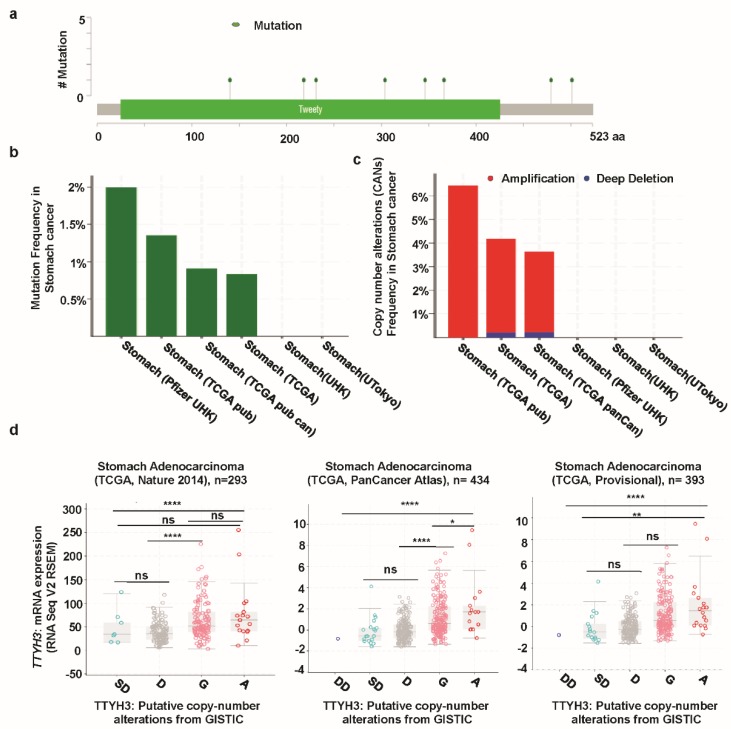
Mutation and copy number alterations in *TTYH3* in GC/SC determined using cBioPortal (http://www.cbioportal.org): (**a**) Alteration of a total of eight mutation spots was detected between amino acids 0 and 523 of the TTYH3 protein. The lollipop plots show the type and location of mutations. (**b**) *TTYH3* mutation frequencies in GC/SC was presented as bar diagram. (**c**) Frequency of genomic alterations in *TTYH3* in GC/SC was presented as bar diagram. (**d**) The graph depicts the correlation between *TTYH3* expression and copy number alterations in GC/SC of TCGA data. Abbreviations represent the types of copy number alterations: deep deletions (DD), shallow deletion (SD), diploid (D), gain (G), and amplification (A). (*p < 0.05; ****p < 0.0001; *ns*, not significant)

We next analyzed mutations and CNAs in *TTYH3* in a cohort of GC patients using cBioPortal web. Eight mutations were identified in the TTYH3 protein; most involved the Tweety domain (Figure 5a). Moreover, the mutation frequencies were approximately 2% and 1% in the Pfizer UHK (University of Hong Kong) and TCGA datasets, respectively (Figure 5b). TCGA data displayed the most CNAs of *TTYH3*. Among the CNAs, amplification was the dominant alteration and was found in approximately 4–6% of the patients (Figure 5C). CNAs in GCs/SCs were significantly correlated with the *TTYH3* expression level in TCGA data (one-way ANOVA analysis, *p* = 0.0001) (Figure 5d). Specifically, amplification and gain were predominantly correlated with *TTYH3* expression. These data suggested that augmented *TTYH3* gene expression could be partly due to CNAs in GC/SC.

### 3.5. Correlation of TTHY3 Expression and Patient Survival in GC/SC

**Figure 6:**
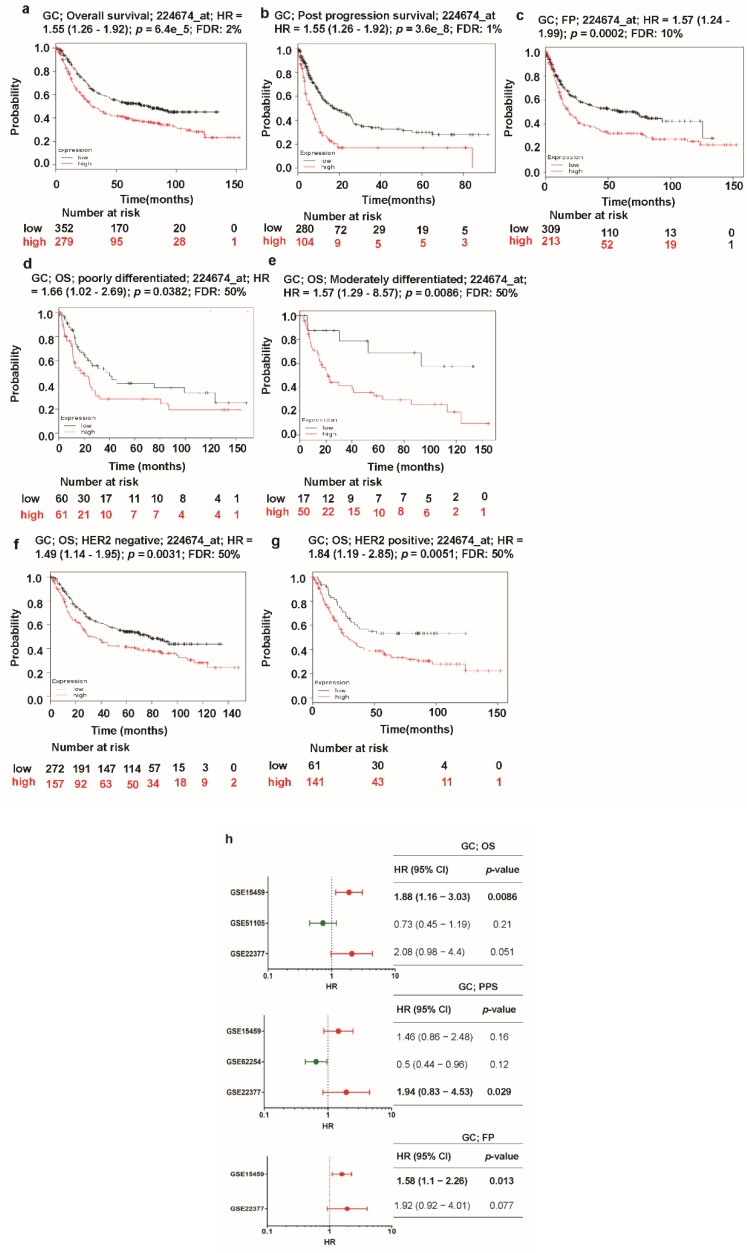
Kaplan–Meier plot of the relationship of *TTYH3* gene expression and survival in GC/SC patients: The survival curves demonstrate patient survival with high (red) and low (black) *TTYH3* expression in GC in Kaplan–Meier plots (http://kmplot.com/analysis/): (**a**) overall survival (OS), (**b**) post-progression survival (PPS), (**c**) first progression survival (FPS), (**d**) overall survival in poorly differentiated GC, (**e**) overall survival in moderately differentiated GC, (**f**) overall survival in HER2 negative GC, and (**g**) overall survival in HER2-positive GC. The analyses focused on the *TTYH3* expression level in GC/SC patients. Cox *p*-value < 0.05. (**h**) Forest plots of GEO datasets evaluating association, *TTHY3*, OS, PPS, and FPS in GC. Hazard ratio (HR) with 95% confidential interval (CI) and *p*-value were labeled in the right column of each forest plot.

Despite the functional role of *TTYH3* in human carcinogenesis, the relationship between *TTYH3* expression and the clinical prognosis of the diseases has not been clarified. Presently, Kaplan–Meier analysis showed the relationship between the expression of *TTYH3* and survival in GC/SC patients with different clinicopathological factors (Figure 6). OS, PPS, and FP were all significantly negatively correlated with *TTYH3* expression in GC/SC patients (Figure 6a–c). OS was also negatively correlated with *TTYH3* expression in subpopulations of patients of poor and moderate differentiation levels or in both HER2 expression positive and negative groups (Figure 6d–g). The meta-analysis was performed to make a relationship between TTYH3 expression and translational clinical relevance using the data from Kaplan–Meier plotter by depicting as forest plots (Figure 6h). Hazard ratio (HR) of OS and FP in GSE15459 and PPS in GSE22377 were significantly higher than 1, showing that higher *TTHY3* expression is correlated with poor clinical outcomes. Moreover, we performed univariate and multivariate analyses for the clinical outcomes regarding *TTYH3* expression in various clinicopathological parameters using Kaplan–Meier plotter web. Univariate analysis showed a significant relationship between *TTYH3* mRNA expression and overall survival in terms of various clinicopathological parameters in GC/SC patients including male; surgery alone; HER2+/-; poorly and moderately differentiation; stages 3 and 4; stages T2 and T3; stages N-1+2+3, N-1, and N-2; and intestinal and mixed lauren classification (Table 2). The multivariate analysis also showed a significant association between *TTYH3* mRNA expression and overall survival in GC/SC patients (Table 2). These analyses confirmed the prognostic relevance of *TTYH3* expression in GC/SC patients.

### 3.6. Analysis of Genes Co-Expressed with TTYH3 in GC/SC 

**Figure 7:**
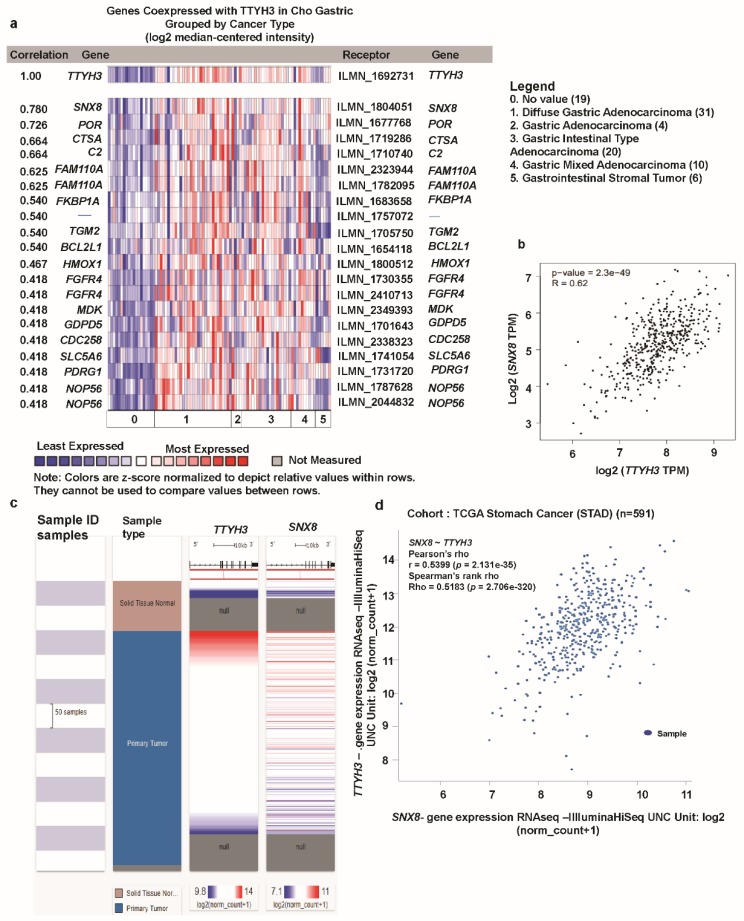
Co-expression profile of the *TTHY3* gene in gastric cancer: (**a**) The co-expression gene profile of *TTYH3* was analyzed using Oncomine. (**b**) The co-expression analysis was performed between *TTYH3* and *SNX8* transcript levels in GC tissues using GEPIA web (http://gepia2.cancer-pku.cn). (**c**) Heat map of *TTYH3* and *SNX8* mRNA expression across gastric cancer in the TCGA database, determined using UCSC (University of California, Santa Cruz) Xena web. (**d**) Co-expression analysis between *TTYH3* and *SNX8* mRNA expression in gastric cancer determined using UCSC Xena web.

Next, we investigated genes that were co-expressed with *TTYH3* in GC/SC using Oncomine and the Cho GC dataset. We identified a set of genes that were positively co-expressed with *TTYH3* in GC/SC (Figure 7a). Among these genes, expression of *SNX8* (*Sorting Nexin 8*) was most highly co-expressed (R = 0.780). Analysis of TCGA data with GEPIA web confirmed the significant positive correlation between *TTYH3* and *SNX8* expression (r = 0.62) (Figure 7b). We finally confirmed this positive correlation of *TTYH3* and *SNX8* using Pearson (r = 0.5399) and Spearman (r = 0.5183) correlation analyses with GC patient TCGA data using the UCSC Xena web (Figure 7c,d). Our findings suggested that expression of *TTYH3* and *SNX8* might be closely corelated and may contribute to a signaling pathway in GC/SC.

### 3.7. Ontology Analysis with TTYH3 and Co-Altered Genes Reveals Signaling Pathways in GC/SC 

**Figure 8:**
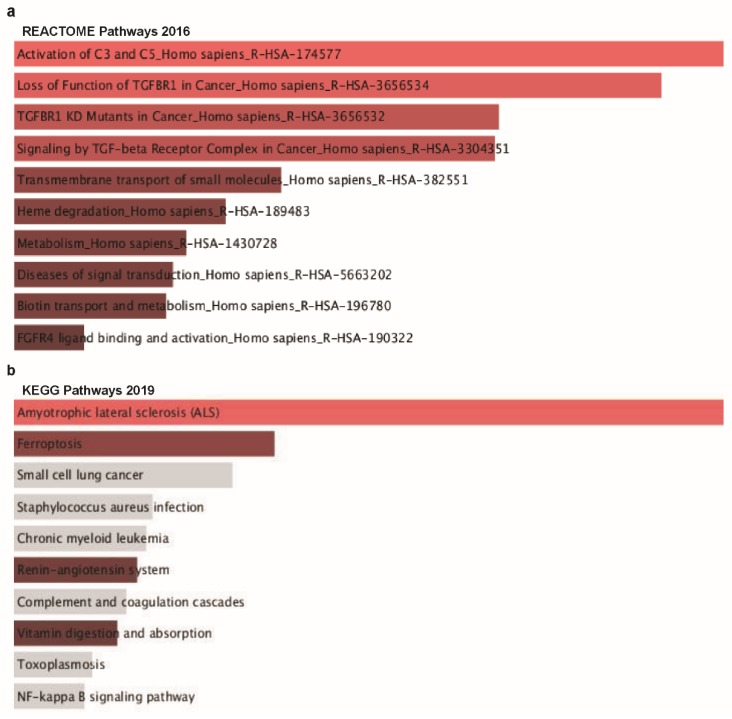

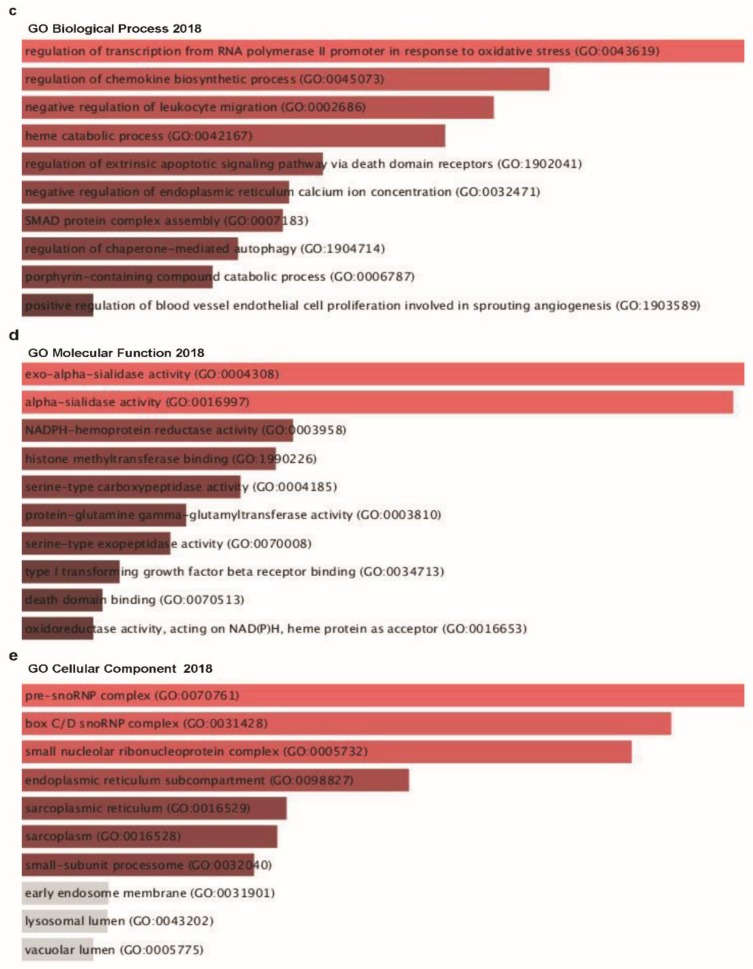
Co-expressed genes profile with the *TTYH3* gene involved in signaling pathways in GC/SC: Enrichr Bar Graph data were collected from Enricher web (https://amp.pharm.mssm.edu/Enrichr). This figure depicts the results showing the gene ontology (GO) and signaling pathways of *TTYH3* and the positively correlated genes in gastric cancer. (**a**) The bar graphs represent genes positively correlated to *TTYH3*, showing the involvement in GC/SC; pathway analysis was done using Enricher (REACTOME pathways 2016). (**b**) Kyoto Encyclopedia of Genes and Genomes (KEGG) pathways 2019. (**c**) Enrichment of GO Biological Process (2018) terms in proteomic analysis. (**d**) Enrichment of GO Molecular Function (2018) terms in proteomic analysis. (**e**) Enrichment of GO Cellular Component (2018) terms in proteomic analysis. The bar graph represents the ratio of the percent composition of terms in proteomic data vs. percent composition in the genome annotation. The length of the bar represents the significance of that specific gene-set or term. The brighter the color, the more significant that term is.

Lastly, to explore our aim of identifying possible signaling pathways from the list of co-altered genes with *TTYH3* in GC/SC, we performed ontology analysis with 16 positively co-altered genes with *TTYH3* obtained from the Oncomine database (Figure 7a). The top 10 REACTOME pathways obtained from the list of *TTYH3* and positively correlated genes were mainly related to the activation of the complement system (C3 and C5), loss of function of *TGFBR1* in cancer, *TGFBR1* kinase domain mutants in cancer, signaling by transforming growth factor-beta receptor complex in cancer, transmembrane transport of small molecules, degradation of heme protein, metabolism, diseases of signal transduction, biotin transport into cells and metabolism, and fibroblast growth factor receptor 4 ligand binding and activation (Figure 8a). Furthermore, the top 10 Kyoto Encyclopedia of Genes and Genomes (KEGG) pathways for *TTYH3* and its positively correlated genes were mainly related to amyotrophic lateral sclerosis, small cell lung cancer, *Staphylococcus aureus* infection, chronic myeloid leukemia, renin-angiotensin system, complement and coagulation cascades, vitamin digestion and absorption, toxoplasmosis, and nuclear factor-kappa-B signaling pathways (Figure 8b). These pathways might be related to tumor development and involved in GC tumorigenesis.

Next, GO analysis was performed with *TTYH3* and its positively correlated genes using the Enrichr tool to analyze functions in biological processes, molecular functions, and cellular components. *TTYH3* and positively correlated genes were mainly related to the regulation of transcription from the RNA polymerase Ⅱ promoter in response to oxidative stress linked with the top biological process (Figure 8c), exo-alpha-sialidase activity in molecular function (Figure 8d), and pre-sno-ribonucleoprotein complex in cellular component (Figure 8e).

## 4. Discussion

GC/SC remains one of the major causes of cancer-related death worldwide [2]. Complete removal by surgery or endoscopic resection is the primary therapeutic technique in early stage GC/SC for the best cure rate [35]. However, the mortality rate remains high for GC/SC patients who are at an advanced stage when diagnosed despite the use of surgery, radiotherapy, and chemotherapy. Novel approaches including targeted therapies based on molecular profiling of GC/SC have improved survival in several cases of advanced GC/SC [36]. The present findings demonstrate that the augmented expression of *TTYH3* was negatively correlated with survival of GC/SC patients, indicating that *TTYH3* could be a therapeutic target for GC/SC.

Gastric cancer/stomach cancer (GC/SC) is highly heterogenous genotypically and phenotypically. For example, it can be categorized morphologically into two types: (i) diffuse and (ii) intestinal types [37]. Diffuse type of GC/SC is clinically more aggressive than the intestinal type which can indicate that the diffuse type shows more metastasis, poorer prognosis, and more resistant to therapy than the intestinal type [38,39,40,41,42]. To improve the accountability and clinical application of the *TTYH3* phenotype, additional datasets might be considered into the analysis, reflecting the heterogeneity feature of GC/SC.

Chloride transport across the plasma membrane of cells regulates cell volume and membrane potential. Chloride channels are involved in trans-epithelial transport and cellular immune responses [43]. The involvement of some chloride channels in tumor progression has been reported in cervical, nasopharyngeal, breast, lung, colon, and pancreatic cancers [44,45,46,47,48,49]. In GC/SC, Ca^2+^-activated Cl^-^ channel markers, including *CL1C1* and *TMEM16A*, are reportedly overexpressed and negatively correlated with patient survival [11,12]. Expression of TMEM16A protein has been negatively correlated with E-cadherin in GC/SC tissues, and knockdown of *TMEM16A* specifically upregulates E-cadherin expression and inhibits migration and invasion specifically in GC cells [12]. Among three human TTYH gene homologs that are the members of Ca^2+^-activated Cl^-^ channel responsive family, only *TTYH2* has been studied in renal cell carcinoma and colorectal cancer [15,16]. *TTYH2* expression is significantly upregulated in the tumors of these cancers and is involved in their proliferation in vitro. Aside from this data, the impact of *TTYH3* on the progress of cancer is unclear.

The present systematic study using bioinformatics analyses of public datasets demonstrates, for the first time, the prognostic value of *TTYH3* in GC/SC. Analyses of the GEO and TCGA datasets revealed the significant upregulation of *TTYH3* in GC/SC tissues and that its expression levels are negatively correlated with the OS and PPS of GC/SC patients. Our data also established the relationship between translational relevance and *TTYH3* mRNA expression in GC/SC patients. The univariate and multivariate analyses also revealed a significant association of *TTYH3* expression with various clinicopathological characteristics in GC/SC patients. Change of gene expression can be caused by genetic mutations, CNAs, and epigenetic control in cancer cells. Further analysis of GC/SC datasets from TCGA revealed a mutation rate of 2%, a 6% amplification in CNAs, and significantly decreased methylation. The TCGA data was used to perform promoter methylation analysis through UALCAN web. Among the normal tissue data in TCGA stomach cancer dataset, only two samples have methylation data. Although two normal samples were too small for comparison with tumor samples, the difference in methylation was statistically significant (*p* = 1E−12). To make clear biological importance, additional data might be used for normal control or independently proved by different methods. Thus, these results imply that increased expression of *TTHY3* in GC/SC could, in part, be caused by one or a combination of these factors. Further studies are needed to more comprehensively explore the detailed molecular mechanisms of this altered biomarker in the progression and prognosis in GC/SC patients.

To explore *TTYH3*-related altered pathways in GC/SC, genes that were co-altered along with *TTYH3* were analyzed. Among the positively correlated genes analyzed in the Oncomine database, *SNX8* expression was most highly co-altered along with *TTYH3* expression. Co-alteration of *SNX8* and *TTYH3* was also confirmed by other analyses in other online platforms. SNX8 is the member of the soring nexin family. It regulates endosome to Golgi transport [50]. Although more than 90% of GC/SC tissues expressed the SNX8 protein, as per the immunohistochemistry results based on data in the Human Protein Atlas (unpublished data), no study has assessed the role of *SNX8* expression in any cancer. We performed pathway analysis with 16 genes that were co-altered along with *TTYH3* to identify pathways related with *TTYH3* expression.

In addition, we utilized Enrichr web tools to determine pathways associated with commonly correlated genes of *TTYH3* in GC/SC. Moreover, from a functional classification viewpoint, *TTYH3* and co-altered genes were subjected in GO enrichment analysis of biological processes, cellular components, and molecular functions. In REACTOME pathway analysis, the most highly correlated pathway was activation of the C3 and C5 complement system in the tumor, which has a role in the regulation of the cancer microenvironment and has been suggested as a target in cancer immunotherapy [51,52,53]. Tumorigenesis is largely affected by the cancer microenvironments, which consist of tumor-associated cells and noncellular component like extracellular matrix [54,55]. Therefore, the signature gene expression of tumor-associated cells in expression profiles of tumor tissues has been suggested as prognostic factor [56,57,58]. The complements, important parts of innate immune system, are mainly expressed from tumor-associated immune cells and play a regulatory role for tumorigenesis [53,59]. Co-alteration of *TTYH3* and a group of the genes involved in the pathway of activation of the C3 and C5 complementary system implies a potential role of *TTYH3* expression modulating cancer microenvironment. The next most correlated pathway was the loss of function of TGFBR1, a protein that is important in various cancers [60]. In the GO analysis of the cellular component domain, the two most enriched ontology terms were related with the complex involving small nucleolar RNA (snoRNA), a small noncoding RNA that associates with ribonucleoproteins (RNPs) to form the stable and functional snoRNP complex [61]. Systematic analysis has shown the elevated expression of snoRNAs in clinical subtypes of multiple cancers [62]. Guangzhong Xu et al. analyzed the altered genes and summarized the mechanism underlying the transcriptional regulatory network in GC/SC [63]. *TTYH3* is one of the DEGs in GC/SC involved in the regulatory network of transcription factor BRCA1 and ZNF263. BRCA1 in the tumor repressor gene and its loss of function are important prognostic factors in GC/SC [64]. *TTYH3* might be involved in BRCA1-related tumorigenesis and GC dissemination mechanism. Further in vitro and in vivo studies are desirable to clarify the biological role of *TTHY3*. These collective findings from the pathway analyses and GO enrichment categories may indicate the important role of *TTYH3* and its co-altered genes in various oncogenic processes.

## 5. Conclusions

In this mining study, we used several online bioinformatic platforms and web tools to systematically analyze the expression, methylation status, mutations and CNAs, correlated genes, and prognostic value of *TTYH3* in human gastric cancer. The multiomics analysis revealed that *TTYH3* is upregulated distinctively and is negatively correlated with clinical outcomes in GC/SC. The elevated expression of *TTYH3* could be regulated through promoter methylation and CNAs. The present findings also reveal the importance of *TTYH3* expression and possible *TTYH3*-related pathways in cancer progression. The findings indicate the potential of *TTYH3* as a therapeutic target for GC/SC.

## Figures and Tables

**Table 1:** The relationship between the *TTYH3* and the clinicopathologic parameters of stomach cancer (TCGA data).

Parameters	*TTYH3*		
	mRNA Expression	# of Sample (*n*)	*p*-Value
**Sample types**			
Normal	↓	34	**1.00E−12**
Primary tumor	↑	415	
**Individual cancer stages**			
Normal	↓	34	
Stage 1	↑	18	**4.60E−04**
Stage 2	↑	123	**1.62E−12**
Stage 3	↑	169	**1.62E−12**
Stage 4	↑	41	**1.98E−11**
**Tumor grade**			
Normal	↓	34	
Grade 1	↑	12	**5.52E−03**
Grade 2	↑	148	**1.00E−12**
Grade 3	↑	246	**1.00E−12**
**Patient’s gender**			
Normal	↓	34	
Male	↑	264	**1.62E−12**
Female	↑	147	**1.11E−16**
**Patient’s age**			
Normal	↓	34	
21–40 Yrs.	↑	4	**4.67E−02**
41–60 Yrs.	↑	128	**1.00E−12**
61–80 Yrs.	↑	253	**1.00E−12**
81–100 Yrs.	↑	25	**1.21E−04**
**Patient’s race**			
Normal	↓	34	
Caucasian	↑	260	**1.00E−12**
African-American	↑	12	**2.36E−03**
Asian	↑	87	**1.05E−14**
**Histological subtypes**			
Normal	↓	34	
Adenocarcinoma not otherwise specified (NOS)	↑	155	**1.00E−12**
Adenocarcinoma Diffuse	↑	69	**1.09E−10**
Adenocarcinoma Signet Ring	↑	12	**9.36E−03**
Intestinal Adenocarcinoma (NOS)	↑	73	**1.00E−12**
Intestinal Adenocarcinoma Tubular	↑	76	**1.00E−12**
Intestinal Adenocarcinoma Mucinous	↑	20	**3.70E−07**
Intestinal Adenocarcinoma Papillary	↑	7	**1.97E−02**
***H. pylori* infection status**			
Normal	↓	34	
With *H*. *pylori* infection	↑		**4.74E−06**
Without *H*. *pylori* infection	↑		**1.62E−12**
Not available	↑		**1.62E−12**
**Additional_surgery_locoregional_procedure**			
Yes		1	ns
No	↑	29	
**Additional_surgery_metastatic_procedure**			
Yes		5	**0.0152**
No	↑	37	
**Additional_pharmaceutical_therapy**			
Yes		29	**0.0092**
No	↑	52	
**Radiation therapy**			
(Discrepancy)		1	
No		366	0.1687
Yes	↑	77	
**Anti-reflux treatment**			
No	↑	202	**0.0145**
Yes		50	

**Table 2:** Correlation of *TTYH3* mRNA expression and clinical prognosis in gastric cancer with different clinicopathological factors by Kaplan–Meier plotter (univariate and multivariate analysis).

Clinicopathological Characteristics		Overall Survival (*n* = 882)			
		Univariate Analysis		Multivariate Analysis	
	*n*	Hazard Ratio	*p*-Value	Hazard Ratio	*p*-Value
**All**	882			HR = 1.55 (1.25−1.92)	**6.4E−05**
**Gender:**					
Female	244	HR = 1.35 (0.84–2.16)	0.21		
Male	567	HR = 1.85 (1.38–2.48)	**3.4E−05**		
**Treatment:**					
Surgery alone	393	HR = 1.66 (1.17−2.35)	**0.0042**		
5-fluorouracil (5-FU) based adjuvant	158	HR = 1.15 (0.98−1.35)	0.086		
Other adjuvants	80	HR = 1.61 (0.56−4.64)	0.37		
**HER2 Status:**					
HER2 Negative	641	HR = 1.49 (1.14−1.95)	**0.0031**		
HER2 Positive	425	HR = 1.84 (1.19−2.85)	**0.0051**		
**Differentiation:**					
Poorly differentiated	166	HR = 1.66 (1.02−2.69)	**0.038**		
Moderately differentiated	67	HR = 3.32 (1.29−8.57)	**0.0086**		
Well differentiated	32	HR = 1142066042.6 (0−Inf)	0.41		
**Stage:**					
Stage 1	69	HR = 3.55 (0.77−16.25)	0.082		
Stage 2	145	HR = 1.51 (0.8−2.86)	0.2		
Stage 3	319	HR = 1.86 (1.28−2.71)	**0.00093**		
Stage 4	152	HR = 1.68 (1.1−2.57)	**0.016**		
**Stage T:**					
Stage T-1	14	HR = 1.15 (0.98−1.35)	0.086		
Stage T-2	253	HR = 1.78 (1.16−2.74)	**0.0076**		
Stage T-3	208	HR = 1.84 (1.27−2.67)	**0.0011**		
Stage T-4	39	HR = 1.92 (0.75−4.91)	0.17		
**Stage N:**					
Stage N-0	76	HR = 1.52 (0.58−3.97)	0.39		
Stage N-1+2+3	437	HR = 1.82 (1.4−2.37)	**6.8E−06**		
Stage N-1	232	HR = 2.13 (1.41−3.21)	**0.00022**		
Stage N-2	129	HR = 1.96 (1.24−3.08)	**0.0031**		
Stage N-3	76	HR = 0.69 (0.39−1.21)	0.19		
**Stage M:**					
Stage M-0	469	HR = 1.6 (1.21−2.11)	**0.00094**		
Stage M-1	58	HR = 2.09 (1.13−3.85)	**0.016**		
**Lauren classification:**					
Intestinal	336	HR = 2.37 (1.63−3.43)	**3.1E−06**		
Diffuse	248	HR = 1.36 (0.93−1.98)	0.11		
Mixed	33	HR = 3.19 (0.95−10.65)	**0.047**		
**Perforation:**					
No	169	HR = 1.56 (0.96−2.52)	0.071		
Yes	4	NA	NA		
